# Predictors of aortic clamp time duration and intensive care unit length of stay in elective adult cardiac surgery

**DOI:** 10.1186/s43044-021-00195-0

**Published:** 2021-10-22

**Authors:** Ashraf Fadel Moh’d, Hayel Talal Al-Odwan, Salah Altarabsheh, Zeid Mohammad Makahleh, Mohammad Abdallah Khasawneh

**Affiliations:** 1 Queen Alia Heart Institute/Jordanian Royal Medical Services, Amman, Jordan; 2 Cardiac Surgery Department, Queen Alia Heart Institute/Jordanian Royal Medical Services, Amman, Jordan

**Keywords:** Aortic cross-clamp, Cardiopulmonary bypass, Ischaemia time, Length of stay

## Abstract

**Background:**

Aortic cross-clamp utilized during cardiac surgery facilitates motionless and bloodless surgical field. However, the duration of clamp time has an inverse effect on early post-operative recovery period. In this study, we sought to examine the predictors of aortic clamp duration and intensive care unit length of stay.

**Results:**

Six hundred and nine adult patients presented for elective cardiac surgery between December 2019 and December 2020 were enrolled. The age of patients ranged from 18 to 82 years (mean 55.62 years, SD ± 12.3 years). Male/female ratio is 4.6:1. Most patients (87.2%) were planned for coronary artery bypass grafting (CABG) and 78 patients (12.8%) for single heart valve procedure. Operative time (OT) ranged from 120 to 402 min and averaged 259.4 min (SD ± 45.9 min). ACC time ranged from 15 to 159 min and averaged 50.56 min (SD ± 19.4 min). Factors associated with significantly longer ACCT were: smoking (OR = 1.89 (95% CI 1.3–2.74), *p* value = 0.01), respiratory disease (OR = 0.48 (95% CI 0.24–0.96), *p* value = 0.039), obesity (OR = 1.76 (95% CI 1.18–2.63), *p* value = 0.005) and AVR (OR = 2.11 (95% CI 1.17–3.83), *p* value = 0.013). Low cardiac output syndrome (LCOS) was observed in 19.2% of patients. Longer than average ACCT was associated with increased use of inotropes (*p* value < 0.001), intra-aortic balloon pump (*p* value < 0.001) and first 24 h post-operative blood loss (*p* value < 0.001). The average intensive care unit length of stay (ICULOS) was 1.64 days (SD ± 1.1 days). Patients' ACCT converged positively and significantly on ICULOS (Beta coefficient = 1.013 (95% CI 1.01–1.015), *p* value < 0.001).

**Conclusion:**

Aortic cross-clamping is a crucial method in cardiac surgery to achieve motionless field; however, prolongation of this method had an incremental risks for the development of low cardiac output syndrome, increased first 24 h post-operative blood loss and longer stay in the intensive care unit.

**Supplementary Information:**

The online version contains supplementary material available at 10.1186/s43044-021-00195-0.

## Background

The modern era of heart surgery utilizing cardiopulmonary bypass (CPB) began in 1954 when Dr Gibbon reported the development of the CPB machine [1, 2]. A year earlier, Dr Bjorn had established the first intensive care unit in Copenhagen in 1953 [3, 4]

During CPB, constant blood flow is delivered to the patient by mechanical pump [5, 6]. To provide a dry, motionless, operative area, a cross-clamp is placed across the ascending aorta preventing blood from entering the heart chambers [7].

Myocardial ischemia after placement of aortic clamp occurs since coronary arteries are not perfused. Strategies are made to protect the heart from ischemia during this most critical period of surgery [8, 9]. However, despite the advances of cardioplegic solutions, hypothermia and cardiac perfusion, aortic cross-clamping remains the main culprit for myocardial insult, which is usually manifested as low cardiac output syndrome (LCOS) occurring in the early post-operative period, necessitating the use of pharmacological and mechanical cardiac support and mandating prolonged intensive care unit stay [10].

Many clinical studies have shown that the durations of ACC and CPB during cardiac surgery are independent predictors of mortality and morbidity; there is no agreement on a “safe limit of time” and authors have suggested different durations of safe (low risk) utilization of CPB and ACC [11–13].

In this study, we sought to determine the predictors of the duration of aortic clamping and the length of ICU bed occupancy.

## Methods

**Fig. 1:**
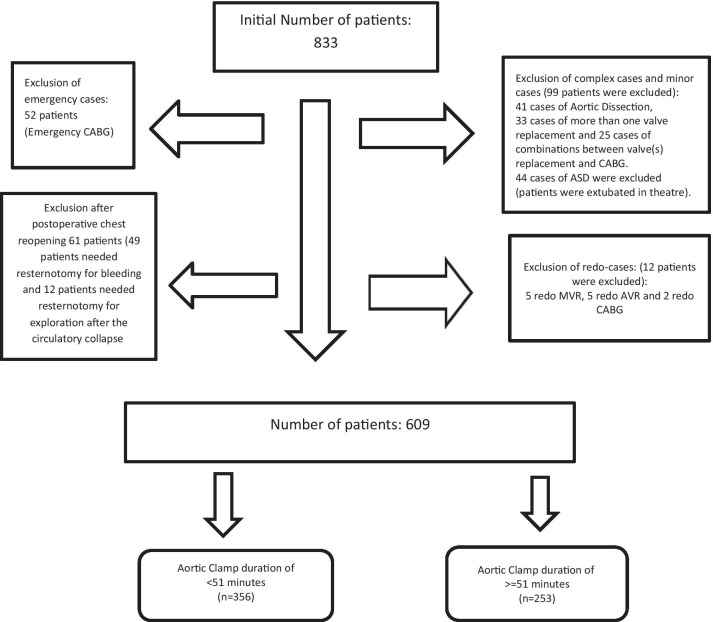
Flow chart of the study

This retrospective observational study analysed data of 609 adult elective cardiac surgical patients who had cardiac surgeries in the period between December 2019 and December 2020. Inclusion criteria included: adult age, elective cardiac surgery, on-pump coronary artery bypass grafting, one heart valve repair or replacement. Exclusion criteria included: OPCAB (off-pump coronary artery bypass, perioperative cardiac arrest and mechanically ventilated before surgery). (More explanation on inclusion and exclusion criteria is given in Fig. 1.)

A unified protocol of cardioplegia is applied in our institution: (1) anterograde cold crystalloid hyperkalaemic type via aortic root and (2) retrograde intermittent cardioplegia through the coronary sinus. All patients received anterograde cold crystalloid cardioplegia with or without retrograde blood-based cardioplegia. Indications for retrograde cardioplegia include moderate to severe aortic regurgitation, severe coronary artery stenosis and anticipated long surgery.

ICU discharge criteria: Patients can be transferred from the ICU to the ward if they fulfil the following criteria: (1) hemodynamically stable, with no inotropic or mechanical support needed, (2) conscious and oriented, (3) with minimal or no chest tube drainage, (4) have adequate respiratory parameters.

Definitions:

Left ventricular impairment: LV impairment was defined by either an echocardiographic assessment or visual estimation of the left ventricular segmental motion by the left heart catheterization or both.

Previous myocardial infarction: Previous myocardial infarction was defined if the patient had any Q wave appearance or enzyme leak in the past 6 months from surgery.

Respiratory disease: This was considered for any patient who is being followed or treated by the pulmonology team.

Smoking status: Any patient who did not quit smoking for the last 6 months from surgery.

Statistical data analysis: The mean and standard deviation were used to describe continuously measured variables, and the frequencies and percentage were applied to categorically measured variables. The multiple dichotomies analysis was used to describe the measured outcomes with more than an option. The Bivariate Spearman's (Rho) test of correlation was used to assess the association between ICULOS with other metric variables. The independent samples t test and the one-way ANOVA test were used to assess the statistical significance of the mean difference of ICULOS across the levels of the categorically measured variables. The Chi-squared test of independence was used to assess the association between categorically measured variables. The multivariate logistic binary regression was used to assess the combined and individual associations between selected predictor independent variables with their odds of having had more than average ACCT. The association between the predictors with the delayed ACCT was expressed as odds ratio (OR) with 95% CI. The generalized linear models multivariate gamma regression was used to regress the ICULOS against clinical and sociodemographic variables. SPSS IBM V21 statistical analysis program was used and the alpha significance level was considered at 0.050 levels. De-identified tables of the study material and the statistical analysis are provided as supplementary materials in the Additional files 1 and 2.

Ethical Committee approval obtained.

## Results

The resulted findings from the descriptive analysis of the patients' sociodemographic, habits and past medical history are displayed in Tables 1, 2.

**Table 1:** Patients' sociodemographic and past medical history characteristics (*N* = 609)

	Number	Percentage	Mean (SD)
*Sex*			
Female	109	17.9	
Male	500	82.1	
Age (years), mean (SD)			55.62 (12.33)
*Age group*			
18–34 years	43	7.1	
35–50 years	106	17.4	
51–65 years	363	59.6	
≥ 66 years	97	15.9	
Body Mass Index score, mean (SD)			26.86 (4.01)
*Body Mass Index score classification*			
Underweight	4	0.7	
Normal	151	24.8	
Preobesity	347	57	
Class I obesity	74	12.2	
Class II obesity	25	4.1	
Class III obesity	8	1.3	
*Smoking habit*			
No	419	68.8	
Yes	190	31.2	
*Comorbid*			
No	258	42.4	
Yes	351	57.6	
*Comorbidity type,* *n* = *351*			
Left ventricular impairment	106	30.2	
CVA	11	3.1	
Hypertension	236	67.2	
Diabetes mellitus	102	29.1	
Obese	107	30.5	
Respiratory illness	43	12.3	
ESRD on dialysis*	1	0.0016	
Moderately impaired renal function**	2	0.003	
Severely impaired renal function*** off dialysis	0	0	
Moderate pulmonary hypertension^†^	2	0.003	
Severe pulmonary hypertension^††^	1	0.0016	
EUROSCORE, mean (SD)			1.01 (0.79)
EUROSCORE group:			
Euroscore < 4 points	598	98.2	
Euroscore ≥ 4 points	11	1.8	

*ESRD: End-stage renal disease, requiring dialysis, regardless of creatinine clearance

**Moderately impaired renal function: creatinine clearance (50-85 ml/min)

***Severely impaired renal function: creatinine clearance (<50 ml/min), not requiring dialysis

^†^Moderate pulmonary hypertension: Pulmonary Artery systolic pressure (31-55 mm Hg)

^††^Severe pulmonary hypertension: Pulmonary Artery systolic pressure (>55mm Hg)

**Table 2:** Descriptive analysis of the patients' cardiac surgical characteristics and outcomes

	Frequency	Percentage Mean (SD)
*Type of underwent cardiac surgery*		
Coronary Artery Bypass Grafting—CABG	531	87.2
Aortic valve replacement—AVR	52	8.5
Mitral valve Replacement—MVR	26	4.3
Total cardiac surgical time (min), mean (SD)		259.39 (45.89)
Cardiopulmonary Bypass time (min), mean (SD)		115.58 (25.03)
Aortic cross-clamp ACCT time (min), mean (SD)		50.58 (19.40)
*Aortic cross-clamp ACCT time group*		
ACCT < 51 min	356	58.5
ACCT ≥ 51 min	253	41.5
Mean body temperature during the operation (degrees Celsius), mean (SD)		33.26 (12.19)
Total Intra ICU Blood Loss (cc) first 24 hours, mean (SD)		499.30 (166.95)
*Required inotropic support*		
No	492	80.8
Yes	117	19.2
*Required intra-aortic balloon pump therapy*		
No	573	94.1
Yes	36	5.9
Intensive Care Unit Length of stay (days), mean (SD)		1.64 (1.1)

Smoking habit was significant predictor for longer ACCT, *p* = 0.002, according to a Chi-squared test of independence, but patients' comorbidity and comorbidity types did not converge significantly on their ACCT, according to the Chi-squared test of association. Although the patients' admission time European cardiac surgery risk score (EUROSCORE) did not differ significantly between the patients who required longer/shorter than average ACCT, *p* = 0.075, the patients who required ACCT ≥ 51 min were found to have measured slightly higher EUROSCORE at admission time on average compared to those patients who had undergone an ACCT of < 51 min.

Type of cardiac surgery did not converge significantly on ACCT according to the Chi-squared test of independence, but the patients who required an ACCT ≥ 51 min indeed stayed longer intraoperatively (mean surgical time = 287 min) on average compared to those who required < 51 min of ACCT (mean operative time = 239.53 min), *p* < 0.001 according to an unpaired samples t test. Also, the patients who required ≥ 51 min ACCT measured significantly longer CPB time (mean = 253.51 min) than those who had < 51 min of ACCT (mean = 102.94 min), *p* < 0.001 according to another unpaired samples t test. As well, a Chi-squared test of independence showed that the patients need for inotropic support in the ICU had correlated significantly with longer ACCT. Indeed, the patients who required extended inotropic support in the ICU were found to be significantly more predicted to have had a longer than average (≥ 51 min) ACCT compared to those who had not required inotropic support in the ICU, *p* < 0.001. Also, the patients' need for intra-aortic balloon pump (IABP) had correlated significantly with longer ACCT; *p* < 0.001. Besides, an independent samples t test showed that the patients who underwent ACCT > 51 min indeed stayed significantly longer periods in the ICU (mean ICULOS = 2.26 days) compared to patients who had less than 51 min of ACCT (ICULOS = 1.21 days) on average, *p* < 0.001 (Table 3).

**Table 3:** Bivariate analysis of the cardiac surgical patients dichotomized aortic cross-clamp time (*N* = 609)

	Aortic cross-clamp time		*p* value
	< 51 min	≥ 51 min	
	*n* = 356	*n* = 253	
*Sex*			
Female	60 (16.9%)	49 (19.4%)	0.425
Male	296 (83.1%)	204 (80.6%)	
Age (years), mean (± SD)	55.22 (± 12.10)	56.20 (± 12.68)	0.335
*Age group*			
Age ≤ 65 years	309 (86.8%)	203 (80.2%)	0.029
Age ≥ 66 years	47 (13.2%)	50 (19.8%)	
Body Mass Index score, mean (SD)	26.63 (± 3.94)	27.17 (± 4.10)	0.107
*Body Mass Index score classification*			
Underweight	4 (1.1%)	0	0.046
Normal	100 (28.1%)	51 (20.2%)	
Pre-obesity	194 (54.5%)	153 (60.5%)	
Class I obesity	42 (11.8%)	32 (12.6%)	
Class II obesity	11 (3.1%)	14 (5.5%)	
Class III obesity	5 (1.4%)	3 (1.2%)	
*Smoking habit*			
No	262 (73.6%)	157 (62.1%)	0.002
Yes	94 (26.4%)	96 (37.9%)	
*Comorbid*			
No	155 (73.5%)	103 (40.7%)	0.486
Yes	201 (56.5%)	15 (59.3%)	
*Comorbidity type* = *351*			
Left ventricular impairment	57 (16%)	49 (19.4%)	0.282
CVA	6 (1.7%)	5 (2%)	0.791
Hypertension	130 (36.5%)	106 (41.9%)	0.179
Diabetes mellitus	60 (16.9%)	42 (16.6%)	0.922
Obesity	58 (16.3%)	49 (19.4%)	0.326
Respiratory illness	28 (7.9%)	15 (5.9%)	0.358
EUROSCORE, mean (SD)	0.96 (± 0.82)	1.10 (± 0.75)	0.075
*Type of underwent cardiac surgery*			
Coronary artery bypass grafting—CABG	316 (88.8%)	215 (85%)	0.169
Aortic valve replacement—AVR	25 (7%)	27 (10.7%)	0.112
Mitral valve replacement—MVR	15 (4.2%)	11 (4.3%)	0.936
Total cardiac surgical time (min), mean (± SD)	239.53 (± 35.54)	287 (± 44.27)	< 0.001
Cardiopulmonary bypass time (min), mean (SD)	102.94 (± 20.64)	253 (± 19.16)	< 0.001
Mean body temperature during the cardiac operation, mean (± SD) degrees Celsius	33.80 (± 15.9)	32.51 (± 1.11)	0.197
Total ICU Blood Loss (cc) first 24 h, mean (SD)	441.22 (± 136.8)	581 (± 171.5)	< 0.001
*Required inotropic support*			
No	334 (93.8%)	158 (62.5%)	< 0.001
Yes	22 (6.2%)	95 (37.5%)	
*Required intra-aortic balloon pump therapy*			
No	334 (93.8%)	158 (62.5%)	< 0.001
Yes	22 (6.2%)	95 (37.6%)	
Intensive care unit length of stay days, mean (SD)	1.21 (± 0.52)	2.26 (± 2.26)	< 0.001

The bivariate analysis of the cardiac patients' ACCT was followed by a multivariate analysis using the binary logistic regression to ascertain the findings (Table 4).

**Table 4:** Multivariate logistic binary regression analysis of the cardiac patients' odds of prolonged aortic cross-clamp time (*N* = 609)

	Multivariate adjusted odds ratio	95% CI for OR		*p* value
		Lower	Upper	
Sex = male	0.718	0.460	1.120	0.144
Age > 65 years	1.717	0.991	2.974	0.054
Smoker = yes	1.889	1.301	2.743	0.001
Body mass type: obese or overweight	1.764	1.182	2.631	0.005
A positive history of respiratory illness	0.484	0.243	0.964	0.039
Underwent AVR surgery = Yes	2.117	1.170	3.829	0.013
EUROSCORE	1.026	0.792	1.330	0.844
Constant	0.435			0.003

Dependent variables = aortic cross-clamp time > 51 min. Model overall statistical significance *χ*^2^(7) = 30.81, *p* < 0.001. Model AUC/ROC = 0.70

The yielded multivariate logistic regression analysis (Table 4) was statistically significant overall, *χ*^2^(7) = 30.81, *p* < 0.001, indicating that at least one or more of the tested patient predictor independent variables had a statistically significant association with their odds of having had prolonged ACCT over 51 min, and to unravel on the main findings, the analysis findings showed that the patients' sex did not converge significantly on their odds of prolonged ACCT, *p* = 0.144, accounting for the other predictor variables in the analysis model. But the analysis model showed that patients aged > 65 years were found to be slightly more (OR: 1.72) predicted for prolonged ACCT, though not statistically significant, compared to patients aged ≤ 65 years on average, *p* = 0.054. Moreover, the analysis model showed that smokers were found to be statistically significantly more predicted (OR: 1.89) for prolonged ACCT compared to non-smoker patients, *p* = 0.001. Interestingly, the analysis model showed that the patients BMI had converged significantly on greater odds of longer than average ACCT; pre-obese and obese patients were found to be significantly more predicted (OR: 1.76) for prolonged ACCT above average compared to the patients with normal and underweight body mass, *p* = 0.005 (Table 5).

**Table 5:** Bivariate Spearman's Rho correlations between the patients ICULOS and their other clinical outcomes and measures

	ICULOS (days)
Patients age in years	0.172**
Total surgical time (min)	0.484**
Cardiopulmonary bypass time (min) (CPBT)	0.562**
Aortic cross-clamp time (ACCT)	0.758**
Intraoperative body temperature (Celsius)	− 0.017
Bleeding amount-blood loss-in (cc) in the ICU	0.599**
EUROSCORE	0.226**

**p value is ≤ 0.010, *p value ≤ 0.050 (*p* values are two tailed)

To understand better what may explain why patients stay longer or shorter in the ICU after cardiac surgery, the patients' mean ICULOS was analysed further for statistically significant mean differences across the levels of the patients measured categorical factors using the bivariate analysis methods. The resulted findings, Table 6, show that male and female patients did not differ significantly on their mean ICULOS, *p* = 0.857, according to an independent samples t test. The patients' grouped age had converged significantly on the ICULOS; patients older than 65 years stayed significantly longer in the ICU than those who are aged 65 years or less, according to an independent samples t test, *p* < 0.001.

**Table 6:** Bivariate analysis of the cardiac surgical patients ICULOS (days) (*N* = 609)

	Mean (SD)-ICULOS (days)	*p* value
*Sex*		
Female	1.66 (1.25)	0.857
Male	1.64 (1.04)	
*Age group*		
Age ≤ 65 years	1.53 (0.93)	< 0.001
Age ≥ 66 years	2.26 (1.51)	
*Body Mass Index score classification*		0.003
Underweight	1 (0.1)	
Normal	1.43 (0.82)	
Pre-obesity	1.73 (1.10)	
Class I Obesity	1.50 (1.01)	
Class II Obesity	2.20 (1.78)	
Class III Obesity	1.75 (1.17)	
*Smoking habit*		
No	1.60 (1.1)	0.109
Yes	1.75 (1.10)	
*Comorbid*		
No	1.47 (0.90)	< 0.001
Yes	1.77 (1.18)	
*Comorbidity type, n* = 351		
Left ventricular impairment		
No	1.53 (0.944)	< 0.001
Yes	2.17 (1.50)	
CVA		
No	1.64 (1.10)	0.164
Yes	2.10 (1.58)	
Hypertension		
No	1.51 (0.95)	< 0.001
Yes	1.86 (1.22)	
Diabetes mellitus		
No	1.56 (0.97)	0.001
Yes	2.10 (1.43)	
Obesity		
No	1.64 (1.03)	0.757
Yes	1.67 (1.27)	
Respiratory illness		
No	1.63 (1.10)	0.282
Yes	1.81 (1.12)	
*Type of underwent cardiac surgery*		
Coronary artery bypass grafting—CABG		
No	1.56 (0.96)	0.485
Yes	1.66 (1.10)	
Aortic valve replacement—AVR		
No	1.65 (1.10)	0.843
Yes	1.62 (0.99)	
Mitral valve replacement—MVR		
No	1.65 (1.08)	0.378
Yes	1.64 (0.91)	
*Required inotropic support*		
No	1.37 (0.71)	< 0.001
Yes	2.80 (1.50)	
*Required intra-aortic balloon pump therapy*		
No	1.54 (0.92)	< 0.001
Yes	3.33 (1.74)	

*p* values are two tailed

The multivariate gamma regression analysis (Table 7) showed that patient's sex did not correlate significantly with their ICULOS, *p* = 0.463, but their age had correlated significantly and positively with their ICULOS. It is predicted that as patients age rise by one year on average their ICULOS days rise by a factor equal to 0.1% times more on average, *p* < 0.001, accounting for the other predictors in the analysis model. Also, the patients who required inotropic support in the ICU stayed significantly longer (22.3% times more) compared to those who did not require Inotropic supportive agents, *p* < 0.001. However, the patients' BMI (obesity and pre-obesity) did not converge significantly on their ICULOS, considering the other predictors in the analysis, *p* = 0.347. But, the multivariate analysis model suggested that the patients' ACCT had converged significantly and positively on their ICULOS, *p* < 0.001, for each additional 1 min rise in the patients' ACCT their corresponding ICULOS tended to rise by 1.3% times more on average, considering the other predictors in the analysis as accounted for, however. Likewise, the patients' post-operative first 24-h blood loss had correlated significantly and positively on their ICULOS, for each additional one cubic centimetres of blood loss the patients' ICULOS is predicted to rise by 0.1% times more on average, *p* < 0.001. Not only so, but the patients EUROSCORE had correlated significantly and positively with their ICULOS, for each additional one point rise in the EUROSCORE their predicted ICULOS days tended to rise by a factor equal to 4.3% times higher on average, *p* = 0.046, well by considering the other predictor variables in the analysis model as accounted for.

**Table 7:** Multivariate generalized linear gamma regression explaining the cardiac patients' length of intensive care unit stay (days)

Parameter	Exponentiated beta coefficient	95% Wald C. I for Exp(B)		*p* value
		Lower	Upper	
(Intercept)	0.414	0.350	0.490	< 0.001
Sex = male	1.028	0.955	1.105	0.463
Age (years)	1.005	1.002	1.007	< 0.001
Required inotropic support in the ITU/OR	1.223	1.124	1.330	< 0.001
Body mass type: obese or overweight	1.032	0.967	1.101	0.347
Aortic cross-clamp time in minutes	1.013	1.010	1.015	< 0.001
First 24 h total blood loss (cc)	1.001	1.000	1.001	< 0.001
EUROSCORE	1.043	1.001	1.086	0.046

Dependent variable: ICU length of stay days. Estimating method = gamma regression with maximum likelihood. Exponentiated beta coefficient is interpreted just like a risk rate or like an odds ratio

## Discussion

While several risk stratification models and algorithms take into consideration pre-operative patient factors (EUROSCORE II (The European System for Cardiac Operative Risk Evaluation II), STS (society of thoracic surgery) score, Parsonnet score, ACEF score (an acronym for age, pre-operative creatinine, and ejection fraction)), scoring systems that take into consideration important intraoperative factors such as ACCT, CPBT are non-existent to guide post-operative patient care [14, 15].

Results of our study showed that longer durations of ACCT were associated with increased ICULOS, post-operative bleeding, need for inotropes and use of IABP support. We also found several risk factors for prolonged ACCT such as smoking status, respiratory disease, obesity and AVR surgery. Several studies examined the impact of ACCT on patients' outcomes. Iino et al. reported that in 16,272 patients with aortic valve replacement, prolonged ACCT was independent predictor of post-operative morbidity and mortality [16]. Al-Sarraf report demonstrated that in 3799 consecutive patients who had cardiac surgery that prolonged ACCT significantly correlates with major post-operative morbidity and mortality in both low- and high-risk patients, especially in those who had ACCT > 90 min [17]. Shultz et al. studied the impact of longer ACC durations (more than 300 min) on complex cardiac surgeries and found that cardiac procedures requiring extremely long ischemic times have significant early mortality and morbidity [18]. Other studies explained the mechanism of myocardial injury after longer ACC durations; Erkut et al. investigated 51 patients scheduled for elective coronary artery bypass grafting (CABG) by taking three troponin I measurements for each patient at different stages and found that there is a direct and linear correlation between ACCT and post-operative troponin I levels [19]. Moreover, Erkut et al. suggested a 50 min. threshold as a safety limit for an ACCT in elective CABG [19]. On the other hand, the impact of prolonged CPB on patients' recovery is explained by another mechanism, which is mainly related to the positive correlation with the interleukin-6 response that is responsible for the systemic inflammatory response (SIR) associated with adverse outcomes in cardiac surgery [20, 21].

Different studies have suggested different safe (low risk) time limits of ACC during on-pump heart surgery. Bar-El et al. reported 2-h ACCT for complex procedures to be safe with retrograde hyperkalaemic blood-based cardioplegia [22]. Erkut reported 50 min ACCT to be safe with cold crystalloid cardioplegic solutions [19], and Al-Sarraf suggested ACCT of less than 60 min to be of low risk [17]. “Safe” ACC duration is mainly related to adequacy of myocardial preservation during this limit of time and importance are factors such as type, root and mode of cardioplegic solution [23].

In our study, the average ACCT was dichotomized based on the average ACCT for the sample (mean ACCT = 51 min approximately), and this dichotomized value of ACCT was used for the bivariate and multivariate analysis because it characterizes the average ACCT for our centre/setup where these data were measured.

To study the impact of ACCT on patients' outcome, we aimed to include a homogenous study population in terms of the average expected duration of the ACCT by exclusion of relatively simple cases (such as closure of an atrial septal defect, as patients are extubated in theatre and their ICULOS is only for several hours) and the extremely complex cases (such as Bentall procedures, or more than one valve replacement or surgeries combining CABG and valves replacement). The rationale for exclusion of uregnt and emergency cases from this study was due to the fact that higher incidences of delay in ICU discharge were atributed to increased blood loss and chest reopenings after recent intake of clopidogrel (an antiplatelet drug which is given hours before left heart catheterization).

Results of our study showed that the average ICULOS was 1.6 days (SD ± 1.1). Our analysis also revealed that for each 1-min delay above the average ACCT (51 min), there was a corresponding prolongation of ICULOS of 1.3% above the average. ICU discharge to a lower dependency unit or surgical ward is a critical period of recovery after open-heart surgery. It is often used as a yardstick for recovery in busy cardiac centres especially in the era of fast-track cardiac anaesthesia [24–26]. The relation between ACCT and ICULOS was investigated by several studies. Cislaghi et al. investigated an audit of 5123 patients and concluded that longer ACCT was associated with a greater likelihood of prolonged post-operative mechanical ventilation and hospital stay [27]. Kapadohos investigated perioperative factors that prolong the ICULOS after open-heart surgery and found that ACCT (along with other factors were strongly related with prolonged ICULOS [28]. Prolonged ICU stay is an important predictor of adverse immediate, short-term and long-term outcomes after cardiac operations [29, 30]. Hassan et al. studied 3,478 cardiac surgical cases and found that patients who had prolonged ICULOS after cardiac operations have worse overall outcomes and experienced higher in-hospital mortality (*p* < 0.0001) [31].

Our analysis showed that for each one millilitre of blood loss post-operatively above the average, the ICULOS increased by 0.1% above average. Bleeding post-cardiac surgery is a well-known factor that causes delay in ICU discharge and exhaustion of hospital resources. Another finding in our study is that for each additional (above average) one-point rise in the EUROSCORE, the predicted ICULOS tended to rise by a factor equal to 4.3% times higher on average. EUROSCORE was a significant predictor of the ICULOS in this study. This can be explained by the fact that EUROSCORE looks on many patient-related factors (age, gender and comorbidities), cardiac related factors (functional classes, left ventricular function, recent myocardial infarction and pulmonary hypertension) and operation related factors (urgency, weight of the intervention and surgery on thoracic aorta).

## Limitations

The main limitations of this study are its retrospective design and that it is a single-centre study. Although we investigated more than twenty variables that may affect the duration of aortic clamping and the ICU discharge after cardiac surgery, many other factors still may play role.

## Conclusions

Our study demonstrated that longer ACCT duration is associated with higher incidence of inotropic support, higher rates of utilization of intra-aortic balloon pump after cardiac surgery and yielded longer stay in the intensive care units. Predictors of longer ACCT included smoking, obesity, pulmonary disease and aortic valve replacement.

## Supplementary Information

**Additional file 1**. Detailed statistical analysis of demographic, clinical and intra-operative factors contributing to the duration of aortic cross-clamp time and post-operative intensive care unit length of stay.

**Additional file 2**. De-identified patients’ data including demographics, co-morbidities, European System for Cardiac Operative Risk Evaluation (EuroSCORE) II, surgical characteristics and intensive care unit time (ICUT)

## Data Availability

Raw data and materials are attached as Additional files 1, 2.

## Abbreviations

ACC: Aortic cross-clamp
ACCT: Aortic cross-clamp time
CPB: Cardiopulmonary bypass
CABG: Coronary artery bypass grafting
ICULOS: Intensive care unit length of stay
LCOS: Low cardiac output syndrome
LOS: Length of stay
OT: Operation time
